# Is laminoplasty or laminectomy the best strategy for C_3_ segment in French-door laminoplasty? A systematic review and meta-analysis

**DOI:** 10.1186/s13018-021-02596-y

**Published:** 2021-09-14

**Authors:** Tiantian Chen, Xun Zhang, Fanchao Meng, Jinglong Yan, Gongping Xu, Wei Zhao

**Affiliations:** Department of Orthopedics, The Second Affiliated Hospital of Harbin Medical University, 148 Baojian Road, Harbin, 150081 China

**Keywords:** French-door laminoplasty, C_3_ laminectomy, C_3_ laminoplasty, Multisegmental cervical spondylotic myelopathy, Meta-analysis

## Abstract

**Background:**

To compare the clinical outcomes of C_3_ laminectomy and C_3_ laminoplasty at the C_3_ segment during French-door laminoplasty.

**Methods:**

The Cochrane Library, PubMed, Embase, and Web of Science databases were searched from inception to November 10, 2020 for studies comparing the clinical outcomes of two types of French-door laminoplasty in the treatment of multilevel cervical spondylotic myelopathy (MCSM). Review Manager 5.3 was used to analyze the following outcomes: operative time, intraoperative blood loss, preoperative and postoperative Japanese Orthopaedic Association (JOA) scores, recovery rate, cervical curvature, cervical range of motion (ROM), incidence of axial symptoms (AS), and C_2-3_ bony fusion rate.

**Results:**

A total of eight studies involving 776 patients were included; there were 424 patients in the C_3_ laminectomy group and 352 patients in the C_3_ laminoplasty group. The results of the meta-analysis showed that the C_3_ laminectomy group was superior to the C_3_ laminoplasty group in terms of operative time (*P* < 0.00001), cervical ROM (*P* = 0.04), and incidence of AS (*P* < 0.0001). However, no statistically significant differences between the two groups were noted regarding intraoperative bleeding (*P* = 0.44), preoperative JOA score (*P* = 0.57), postoperative JOA score (*P* = 0.09), recovery rate (*P* = 0.25), cervical curvature (*P* = 0.22), and C_2-3_ bony fusion rate (*P* = 0.06).

**Conclusion:**

This meta-analysis demonstrated that both C_3_ laminoplasty and C_3_ laminectomy could effectively improve neurological function in patients with MCSM in French-door laminoplasty. However, C_3_ laminectomy can reduce the operative time, preserve cervical ROM, and reduce the incidence of postoperative AS.

**Trial registration:**

PROSPERO registration number is CRD42021230798.

*Date of registration:* February 11, 2021.

## Introduction

Since Kurokawa et al. [1] first reported double-door laminoplasty in 1981, this technique has been continuously improved and is widely used in the treatment of posterior longitudinal ligament ossification (OPLL), developmental cervical spinal stenosis, and multilevel cervical spondylotic myelopathy (MCSM) and has achieved satisfactory results [2]. However, it is difficult to preserve the semispinalis cervicis (SSC) at the C_2_ spinous process while opening the C_3_ lamina in traditional French-door laminoplasty. To completely expose the C_3_ lamina, SSC insertion in C_2_ has been entirely or mostly detached from the C_2_ spinous process and then repaired to the C_2_ spinous process when closing the wound, typically leading to complications, such as a decreased cervical range of motion (ROM), loss of cervical lordosis, and postoperative axial symptoms (AS) [3–5].

To avoid the above problems, conventional French-door laminoplasty has been modified, namely, C_4_–C_7_ laminoplasty with C_3_ laminectomy, and has been widely used in the treatment of MCSM [6]. C_3_ laminectomy theoretically decompresses the spinal cord more adequately, prevents C_3_ lifting from squeezing, stimulates C_2_-attached muscles, and facilitates good neurological decompression while better maintaining the integrity of the structure and function of SSC. The postoperative cervical ROM and physiological curvature can also be better maintained, reducing postoperative AS incidence.

However, the sample size of individual studies comparing the efficacy of modified C_3_ laminectomy and traditional French-door laminoplasty is limited. Objective evaluations of the advantages and disadvantages associated with the two procedures are lacking. Therefore, it is unclear which surgical method can achieve better clinical outcomes. Therefore, we conducted a meta-analysis to compare the clinical and radiological outcomes of these two procedures.

## Methods

### Search strategy

Two researchers searched the Cochrane Library, PubMed, Embase, and Web of Science databases, and the retrieval time was from the inception of these databases to November 10, 2020. The language was not restricted, and the reference list of relevant articles was manually retrieved. The search method can be adapted in different databases. The keywords and their combinations were as follows: (C_3_-C_7_ laminoplasty OR conventional laminoplasty OR bilateral open-door extended laminoplasty) AND (modified cervical double-door laminoplasty OR preserving the semispinalis cervicis OR cervical laminoplasty with C_3_ laminectomy OR C_4_-C_7_ laminoplasty with C_3_ laminectomy OR modified Kurokawa's double-door laminoplasty).

### Inclusion and exclusion criteria of the studies

We adopted the following criteria and studied this meta-analysis: (1) the types of studies included randomized controlled trials, retrospective analyses, or prospective cohort studies; (2) patients with MCSM diagnosed by CT and MRI, including cervical stenosis and OPLL, regardless of sex and race; (3) inclusion in the study included both the "C3 laminectomy group" and the "C3 laminoplasty group"; and (4) the follow-up period was at least 12 months.

The following standards were used to exclude studies: (1) previous cervical surgery for the same disease; (2) nondegenerative diseases, fractures, infections, and intravertebral tumors; (3) studies with duplicate publications, conference abstracts, animal studies, literature reviews, case reports, and biomechanical studies.

### Quality assessment

No randomized controlled trials were included in our research, so the modified Newcastle-Ottawa Scale (NOS) was applied to evaluate the quality of the included studies [7]. Three domains in the NOS were assessed, including the selection of the study population, comparability between groups, and measurement of exposure factors. The quality assessment was conducted independently by two authors, and any disagreement was resolved through discussion until consensus was reached. If debate persists, another reviewer would be invited for a meeting to reach a final agreement.

### Data extraction

Two researchers read the full text carefully and extracted the data independently. Any disagreement was resolved through discussion or by consulting the corresponding authors. The time point of data extraction was the time of the last follow-up, except for the index of AS, which was extracted 3 months after surgery. The effect indicators for data extraction included (1) operative time, (2) intraoperative bleeding, (3) preoperative Japanese Orthopaedic Association (JOA) score, (4) postoperative JOA score, (5) neurological recovery rate, (6) postoperative cervical curvature, (7) postoperative cervical ROM, (8) incidence of AS, and (9) C_2_–C_3_ bony fusion rate.

### Statistical analysis

A meta-analysis of all collected data was performed using the Review Manager 5.3 software provided by the Cochrane International Collaboration. The x^2^ test and I^2^ test were used to evaluate the heterogeneity among studies. When P ≥ 0.1 and I^2^ ≤ 50%, the heterogeneity was considered insignificant, and a fixed effects model combined the data. When the heterogeneity was significant (i.e., P < 0.1 and I^2^ > 50%), the source of heterogeneity was identified to the greatest extent possible, and subgroup analysis or sensitivity analysis was performed. If the source of heterogeneity could not be identified, a random effects model was used for meta-analysis. The odds ratios (ORs) and 95% confidence intervals (CIs) were used to analyze the dichotomous variables' statistics. Weighted mean difference (WMD) and 95% CI were used to combine the mean and standard deviation for continuous variables. *P* < 0.05 was considered statistically significant for comparisons between the two groups.

## Results

### Literature search

**Fig. 1:**
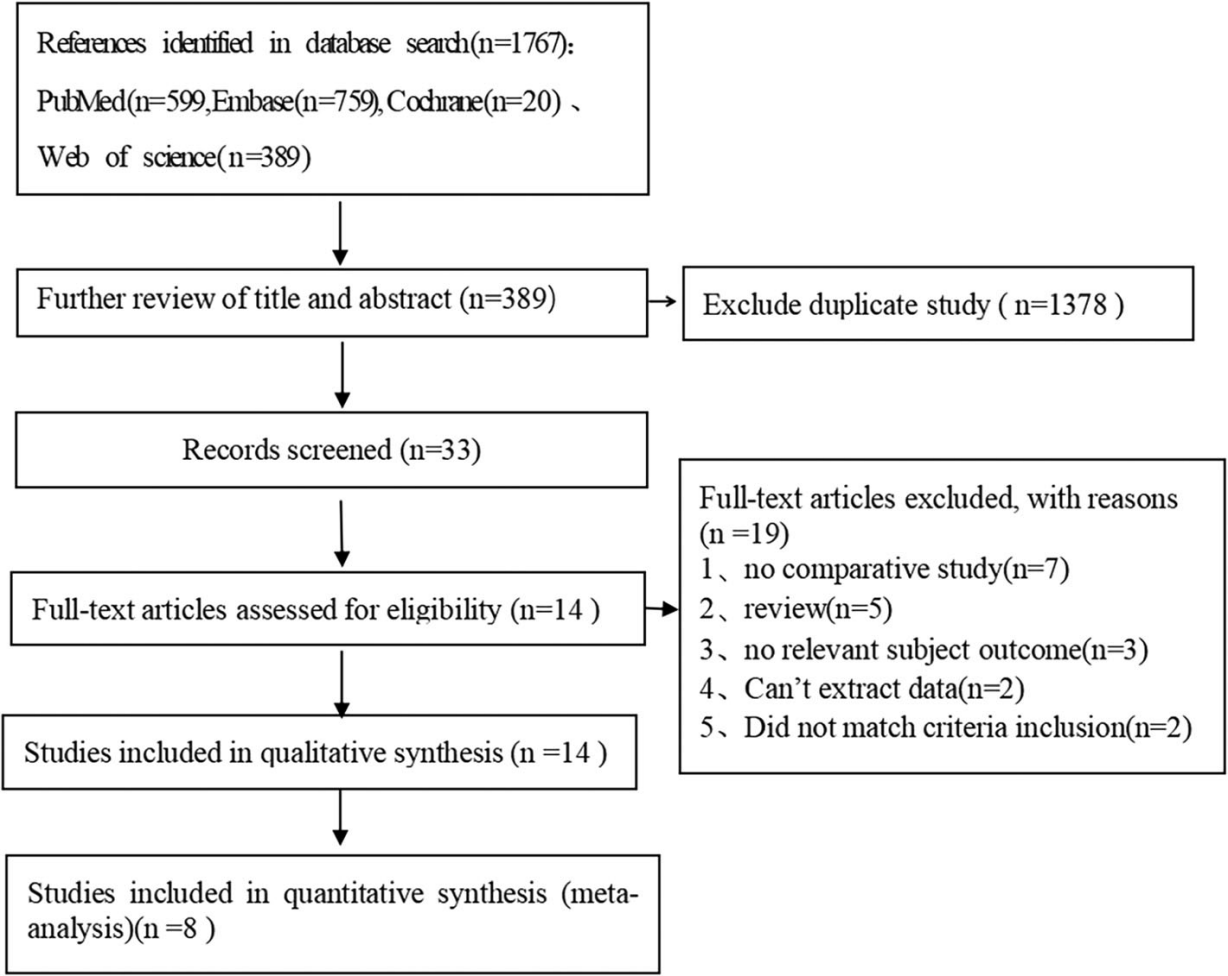
Flow diagram of study selection

As shown in Fig. 1, 1767 publications were initially obtained from the Cochrane Library, PubMed, Embase, and Web of Science. Of those, 1378 studies were duplicates. After reading the titles and abstracts, the studies that did not meet the inclusion criteria were eliminated, and 33 studies were initially screened. Finally, eight studies [6, 8–14] were finally included in the meta-analysis by reading the full text. Among them, the surgical levels of 7 studies were C_3_–C_7_ [6, 8–13], and one study was C_3_–C_6/7_ [14]. A total of 424 patients who underwent C_3_ laminectomy were compared with 352 patients who underwent C_3_ laminoplasty. The data were extracted from the included literature. The primary demographic and clinical characteristics of the included studies are shown in Table 1, and the quality evaluation of the included studies is summarized in Table 2.

**Table 1:** Characteristics of included studies

Author(years)	Country	Study type	Number of samples TG/MG	Gender (male) TG/MG	AgeTG/MG	Follow-up (months) TG/MG	Outcomes
Kudo et al. (2020) [6]	Japan	Retrospective	24/11	17/7	56.3±8.5 56.6±9.6	120 120	1,2,8
Takeuchi et al. (2005) [8]	Japan	Prospective	38/18	27/11	63 (26-90) 59 (46-76)	17 (12-27) 30 (12-42)	3,4,9
Long et al. (2006) [9]	China	Retrospective	36/24	16/20	51 (44-83) 48 (42-81)	12 12	1,2,3,4
Wang et al. (2013) [10]	China	Retrospective	126/46	75/51	58±20 56±18	52±33 52±33	1,2,3,4,5,7,9
Ding et al. (2009) [11]	China	Retrospective	25/13	14/11	65.7 (45-83) 59.0 (46-75)	18 (12-27) 25 (12-50)	3,4,6,7,9
Wang et al. (2015) [12]	China	Prospective	113/39	67/46	58±20 56±18	52±33 52±33	1,2,3,4,5,7,9
Takeuchi et al. (2007) [13]	Japan	Retrospective	80/31	52/28	59.2±11.5 58.9±11.0	19.4±6.0 29.9±11.6	3,4,7
Nakajima et al. (2020) [14]	Japan	Retrospective	106/46	39/67	72.2±9.9 69.1±12.3	12 12	1,2,3,4,5,6,7,8

Outcomes: 1. Operating time; 2. Blood loss; 3. Preoperative JOA score; 4 .Postoperative JOA score ;5. Recovery rate; 6. Cervical lordosis; 7. Intervertebral Range of Motion; 8. The presence of interlaminar bony fusion at C_2_–C_3_. MG : modified French-door laminoplasty group;  TG: traditional French-door group

**Table 2:** Quality assessment of cohort studies according to the Newcastle Ottawa Scale (NOS)

Study	Selection	Compaaability	Exposure	Total Score
Kudo et al. [6]	3	2	3	8
Takeuchi et al. [8]	3	2	3	8
Long et al. [9]	3	2	2	7
Wang et al. [10]	3	2	3	8
Ding et al. [11]	3	2	2	7
Wang et al. [12]	3	2	3	8
Takeuchi et al. [13]	3	2	3	8
Nakajima et al. [14]	3	2	3	8

### Meta-analysis results

#### Operation time

**Fig. 2:**
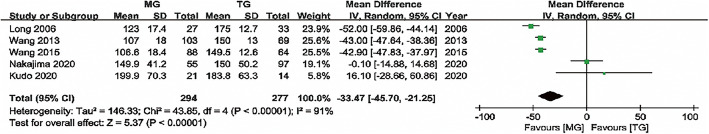
Forest plot showing the weighted mean difference in operative time for MG versus TG

Five studies [6, 9, 10, 12, 14] with 294 and 277 patients compared the operation time between the modified French-door laminoplasty group (MG) and traditional French-door group (TG), respectively. The pooled outcomes showed that the operation time of the MG was shorter than that of the TG (WMD = − 33.47, 95% CI (− 45.70, − 21.25), *P* < 0.00001), and the difference was statistically significant. The random effects model was adopted because of the significant heterogeneity (I^2^ = 91%) (Fig. 2).

#### Intraoperative blood loss

**Fig. 3:**
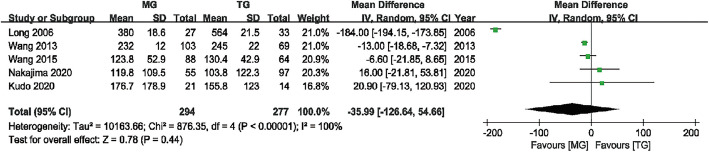
Forest plot showing the weighted mean difference in intraoperative blood loss for MG versus TG

Five studies [6, 9, 10, 12, 14] with 294 and 277 patients compared intraoperative blood loss between the MG and TG, respectively. The pooled outcomes showed no statistically significant difference in intraoperative blood loss between the two groups (WMD = − 35.99, 95% CI (− 126.64, 54.66), P = 0.44). The random effects model was adopted due to the significant heterogeneity (I^2^ = 100%) (Fig. 3).

#### Preoperative JOA score

**Fig. 4:**
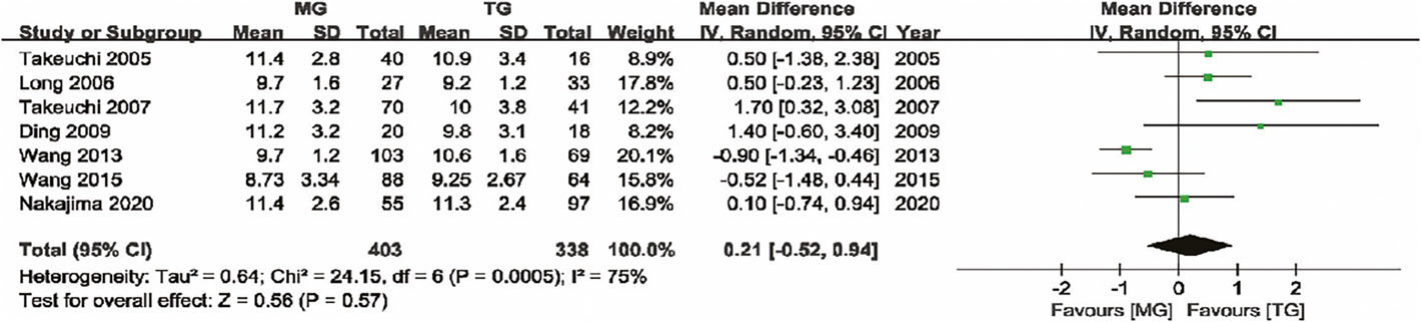
Forest plot showing the weighted mean difference in preoperative JOA score for MG versus TG

Seven studies [8–14] with 403 and 388 patients compared the preoperative JOA scores between the MG and TG, respectively. The pooled outcomes showed no statistically significant difference in preoperative JOA score between the MG and the TG (WMD = 0.21, 95% CI (− 0.52, 0.94) *P* = 0.57), indicating that preoperative neurological function in both groups was similar. The random effects model was adopted due to the significant heterogeneity (I^2^ = 75%) (Fig. 4).

#### Postoperative JOA score

**Fig. 5:**
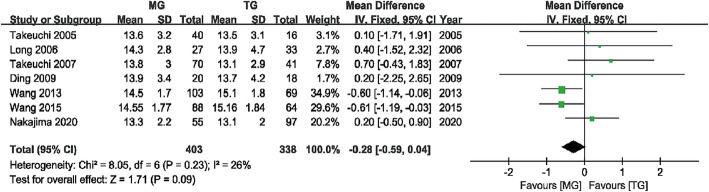
Forest plot showing the weighted mean difference in postoperative JOA score for MG versus TG

Seven studies [8–14] with 403 and 388 patients compared the postoperative JOA scores between the MG and TG, respectively. The pooled outcomes showed no statistically significant difference between the MG and the TG in postoperative JOA score (WMD = − 0.28, 95% CI (− 0.59, 0.04), P = 0.09), suggesting that there was no significant difference in postoperative neurological recovery between the two groups. The fixed effects model was used due to the heterogeneity (I^2^ = 26%) (Fig. 5).

#### Recovery rate

**Fig. 6:**
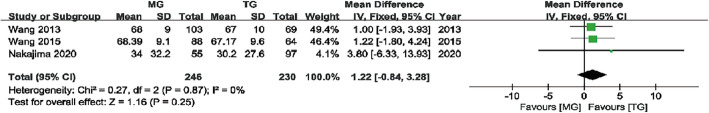
Forest plot showing the weighted mean difference in recovery rate for MG versus TG

Three studies [10, 12, 14] with 246 and 230 patients compared the recovery rate between the MG and TG, respectively. The pooled outcomes showed no statistically significant difference between the MG and TG groups in the recovery rate (WMD = 1.22, 95% CI (− 0.84, 3.28), *P* = 0.25). The fixed effects model was used due to the heterogeneity (I^2^ = 0%) (Fig. 6).

#### Postoperative cervical curvature

**Fig. 7:**
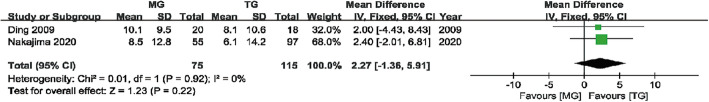
Forest plot showing the weighted mean difference in postoperative cervical curvature for MG versus TG

Two studies [11, 14] with 75 and 115 patients compared the cervical curvature between the MG and TG, respectively. The pooled result showed that the cervical curvature of the MG was greater than that of the TG, but the difference was not statistically significant (WMD = 2.27, 95% CI (− 1.36, 5.91), *P* = 0.22). The fixed effects model was used due to the heterogeneity (I^2^ = 0%) (Fig. 7).

#### Postoperative ROM

**Fig. 8:**
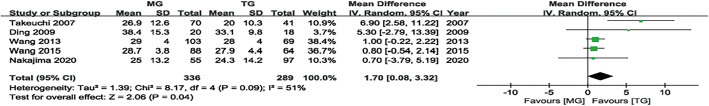
Forest plot showing the weighted mean difference in postoperative ROM for MG versus TG

Five studies [10–14] with 336 and 289 patients compared the postoperative cervical ROM between the MG and TG, respectively. The pooled result showed that the postoperative cervical ROM in the MG was greater than that in the TG with a statistically significant difference (WMD = 1.70, 95% CI (0.08, 3.32), *P* = 0.04). The random effects model was used given the heterogeneity (I^2^ = 51%) (Fig. 8).

#### C_2_–C_3_ Bony fusion rate

**Fig. 9:**
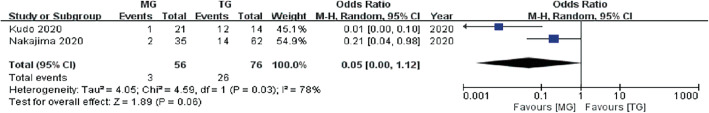
Forest plot showing the odds ratio for C_2_–C_3_ bony fusion rate for MG versus TG

Two studies [6, 14] with 56 and 76 patients compared the C_2_–C_3_ bony fusion rates between the MG and TG, respectively. The pooled result showed that the C_2_–C_3_ bony fusion rate in the MG was greater than that in the TG with a statistically significant difference (WMD = 0.05, 95% CI (0.00, 1.12), *P* = 0.06). The random effects model was used given the heterogeneity (I^2^ = 78%) (Fig. 9).

#### Incidence of axial symptom

**Fig. 10:**
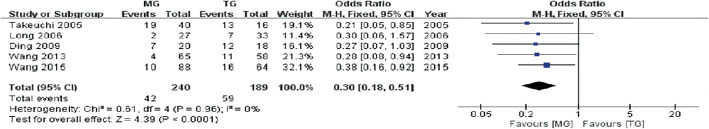
Forest plot showing the odds ratio for incidence of axial symptom for MG versus TG

Five studies [8–12] with 240 and 189 patients compared the incidence of AS between MG and TG, respectively. The pooled result showed that the incidence of AS in the MG was greater than that in the TG with a statistically significant difference (WMD = 0.30, 95% CI (0.18, 0.51), *P* < 0.0001). The fixed effects model was used given the heterogeneity (I^2^ = 0%) (Fig. 10).

## Discussion

Posterior cervical French-door laminoplasty can be used for the treatment of MCSM because it not only directly decompresses compression from the posterior spinal cord but also indirectly decompresses compression from the anterior spinal cord by drifting the spinal cord posteriorly while enlarging the cervical spinal canal. The SSC, which starts at the upper thoracic transverse process and ends mainly at the C_2_ spinous process, is a crucial structure for maintaining the dynamic stability of the cervical spine as an extensor [15, 16]. However, to completely expose the C_3_ lamina during traditional French-door laminoplasty, it is typically necessary to completely or primarily detach the SSC insertion from the C_2_ spinous process and then repair it when the incision is closed to restore the normal biomechanical function of the posterior extensor musculature. However, it was found that 18% of the patients in whom SSC insertion into the C_2_ spinous process had been repaired still had poor functional recovery of the extensor musculature [17]. The attachment site of the SSC varies significantly due to the size and opening angle of the spinous process, so the anatomical location is often difficult to reach by suture and reconstruction, which may be a fundamental reason for the poor postoperative repair of the SSC [18]. The destruction of SSC often leads to the loss of cervical physiological lordosis, the reduction of cervical ROM, and the initiation or aggravation of postoperative AS, which seriously affects the surgical effect. To solve the above complications, researchers invented the modified French-door laminoplasty, in which the SSC was preserved for attachment to the C_2_ spinous process while C_3_ laminectomy was performed [8].

Although modified French-door laminoplasty has been widely used in the treatment of MCSM, which surgical method could achieve better clinical outcomes remains controversial. Therefore, we conducted this meta-analysis. A total of eight articles were included in our meta-analysis. We used the NOS to evaluate the included studies, and the results showed that the quality was high. The results of this meta-analysis indicated that MG was significantly better than TG in terms of operative time, postoperative cervical ROM, and AS incidence. However, no significant differences in intraoperative blood loss, preoperative JOA score, postoperative JOA score, recovery rate, postoperative cervical curvature, or C_2-3_ bony fusion rate were noted between the two groups.

According to our study, the operation time in the MG was shorter than that in the TG. Wang et al. [12] found that the operation times of the MG and the TG were 106.6 ± 18.4 min and 149.5 ± 12.6 min, respectively, and the MG significantly reduced the operation time. However, Nakajima et al. [14] found that there was no statistically significant difference in surgical time between the MG and TG (144.9 ± 41.2 min vs. 150.0 ± 50.2 min). The surgeon's technique may mainly determine the operation time. In general, C_3_ laminectomy omits the separation and reconstruction of the C_2_ spinous process from the attachment muscle, thus reducing the operative time. The operation time is also an essential factor affecting the patient's prognosis. The longer the operation time, the more severe the posterior extensor musculature ischemia and the worse the posterior neck extension, which will ultimately affect the curvature and cervical ROM [9].

AS is common after cervical laminoplasty with an incidence of up to 60% [3]. Patients typically present with neck and shoulder soreness, pain, stiffness, and movement limitations, which significantly reduce their quality of life. Our study showed that the incidence of AS in MG was substantially lower than that in TG, and the difference was statistically significant. The cause of AS remains unclear. Nevertheless, many scholars believe that it is related to the destruction and effect of surgery on posterior cervical extensor musculatures, especially SSC [6, 19, 20]. When it is damaged, the posterior cervical extensor musculatures will continue to compensate for the contraction and shortening of the force arm to maintain balance. Eventually, fatigue and pain will occur, causing AS, such as stiffness and pain in the neck. In addition, the duration of neck brace fixation is also closely related to the incidence of AS [21]. Damage to the SSC typically requires the patient to prolong postoperative external cervical brace fixation, resulting in posterior cervical muscle atrophy and soft tissue adhesion in the neck and thus leading to AS. Cheung et al. [22] found that AS was significantly reduced in the group with cervical brace fixation two weeks after laminoplasty compared with the group without cervical brace fixation in a randomized controlled study. Early removal of the cervical brace allows for earlier implementation of rehabilitation functional exercises, reduces the chance of rupture of the SSC during early postoperative functional training, promotes recovery of the posterior cervical extension power muscles, reduces the probability of muscle atrophy and joint/ligament contracture, and therefore reduces the incidence of AS.

The decrease in cervical ROM is another common complication following traditional French-door laminoplasty, and patients undergoing this procedure exhibit a 30 to 70% reduction in postoperative cervical ROM [23]. The reasons remain controversial and may be related to the prolonged use of the cervical brace and the rupture of the posterior extensor musculature [24, 25]. C_3_ laminoplasty is prone to damage the posterior extensor musculature, especially the SSC inserted into the C_2_ spinous process, which damages the normal extension mechanism of the cervical spine, resulting in loss of postoperative cervical lordosis, a decrease in ROM, neck stiffness, and a reduction in cervical function [13, 20].

Interlaminar bony fusion, especially at C_2_–C_3_, is also a significant cause of reduced cervical ROM and AS [26, 27]. The C_2_ spinous process is caudally curved or relatively hypertrophied. As a result, the space between the C_2_ and C_3_ spinous processes is relatively narrow, which leads to bony impingement between the C_2_ and C_3_ spinous processes during cervical movement [28, 29]. In addition, the posterior arch of C_3_ shifts toward the C_2_ spinous process after traditional French-door laminoplasty, further narrowing the space between it and the C_2-3_ spinous process and increasing the chance of the posteriorly shifted bony posterior arch coming into contact with the posterior cervical muscles, especially during neck movement [25]. Thus, interlaminar bony fusion can lead to neck pain, limited motion, reduced ROM, and even cause further spontaneous bony fusion between the C_2_ and C_3_ posterior arches. Therefore, removal of the C_3_ lamina can reduce the incidence of collision and fusion between the C_3_ lamina and C_2_ spinous process during cervical hyperextension, thus better preserving postoperative cervical ROM and decreasing the incidence of AS [25]. In their 1-year follow-up, Lee et al. [30] found that the interlaminar fusion rate of C_2_–C_3_ in the MG was significantly lower than that in the TG (0/27,0% vs. 7/39,18%), AS was reduced, and the cervical ROM was better (44.6 ± 3.2° vs. 33.8 ± 4.8°). These findings also support our point of view. Our study found that although the rate of C_2_–C_3_ bony fusion in the MG was lower than that in the TG, the difference was not statistically significant, which may be due to the small number of publications that included this index in this study.

This study has some limitations due to the influence of methodological quality and the small sample size of the included studies. First, one of the studies included in this meta-analysis had a surgical segment of C_3_–C_6/7_, whereas the range of posterior cervical laminoplasty in clinical practice is generally C_3_–C_7_. Although preserving the C_7_ lamina can also effectively reduce the incidence of AS without affecting neurological recovery and cervical ROM, it may still influence the analysis results. Second, few studies were included in our research, all of which were retrospective cohort studies with a low clinical evidence level. In addition, the included studies had different diagnostic criteria for AS, which increased the bias of the statistical results. Finally, all patients included in our study were from Asian populations, and the conclusions may not be applicable to other populations outside of Asia. Therefore, more long-term, large-sample, multicenter randomized controlled studies are needed to validate this finding and provide strong evidence for evidence-based medicine.

## Conclusions

In conclusion, both C_3_ laminectomy and C_3_ laminoplasty effectively improve neurological function in patients with MCSM in French-door laminoplasty. However, C_3_ laminectomy can significantly reduce the operative time, preserve cervical ROM, and reduce the incidence of AS.

## Data Availability

All data generated or analyzed during this study are included in this published article and its supplementary information files.

## Abbreviations

OPLL: Posterior longitudinal ligament ossification
MCSM: Multilevel cervical spondylotic myelopathy
SSC: Semispinalis cervicis
ROM: Range of motion
AS: Axial symptom
NOS: Newcastle-Ottawa Scale
OR: Odds ratio
CI: Confidence interval
JOA: Japanese Orthopaedic Association
WMD: Weighted mean difference
MG: Modified French-door laminoplasty group
TG: Traditional French-door group
